# The Type IV Secretion System of ICE*Afe*1: Formation of a Conjugative Pilus in *Acidithiobacillus ferrooxidans*

**DOI:** 10.3389/fmicb.2019.00030

**Published:** 2019-02-05

**Authors:** Rodrigo Flores-Ríos, Ana Moya-Beltrán, Claudia Pareja-Barrueto, Mauricio Arenas-Salinas, Sebastián Valenzuela, Omar Orellana, Raquel Quatrini

**Affiliations:** ^1^Fundación Ciencia y Vida, Santiago, Chile; ^2^Programa de Biología Celular y Molecular, Instituto de Ciencias Biomédicas, Facultad de Medicina, Universidad de Chile, Santiago, Chile; ^3^Facultad de Ciencias de la Vida, Universidad Andres Bello, Santiago, Chile; ^4^Centro de Bioinformática y Simulación Molecular, Facultad de Ingeniería, Universidad de Talca, Talca, Chile; ^5^Millennium Nucleus in the Biology of Intestinal Microbiota, Santiago, Chile

**Keywords:** type IV secretion system (T4SS), mobile genetic element (MGE), ICE*Afe*1, *Acidithiobacillus ferrooxidans*, conjugation, conjugative pili

## Abstract

The dispersal of mobile genetic elements and their gene cargo relies on type IV secretion systems (T4SS). In this work the ICE*Afe*1 Tra-type T4SS nanomachine, encoded in the publicly available genome of *Acidithiobacillus ferrooxidans* ATCC 23270^TY^, was characterized in terms of its organization, conservation, expression and mating bridge formation. Twenty-one conjugative genes grouped in four genetic clusters encode the ICE*Afe*1 T4SS, containing all the indispensable functions for the formation and stabilization of the pili and for DNA processing. The clusters’ organization resembles that of other mobile genetic elements (such as plasmids and integrative and conjugative elements–ICEs). Sequence conservation, genetic organization and distribution of the *tra* system in the genomes of other sequenced *Acidithiobacillus* spp. suggests that the ICE*Afe*1 T4SS could mediate the lateral gene transfer between related bacteria. All ICE*Afe*1 T4SS genes are transcriptionally active and expressed from four independent operons. The transcriptional levels of selected marker genes increase in response to Mitomycin C treatment, a DNA damage elicitor that has acknowledged stimulatory effects on excision rates and gene expression of other ICEs, including ICE*Afe*1. Using a tailor-made pilin-antiserum against ICE*Afe*1 T4SS TraA pilin and epifluorescence microscopy, the presence of the conjugative pili on the cell surface of *A. ferrooxidans* could be demonstrated. Additionally, immunodetection assays, by immunogold, allowed the identification of pili-like extracellular structures. Together, the results obtained in this work demonstrate that the ICE*Afe*1 T4SS is phylogenetically conserved within the taxon, is expressed at mRNA and protein levels *in vivo* in the *A. ferrooxidans* type strain, and produces a pili-like structure of extracellular and intercellular localization in this model acidophile, supporting its functionality. Additional efforts will be required to prove conjugation of the ICE*Afe*1 or parts of this element through the cognate T4SS.

## Introduction

Lateral gene transfer of DNA contributes to bacterial adaptation to changing environments and plays an important role in prokaryotic evolution (Dobrindt et al., 2004). The laterally acquired genes form discrete blocks referred to globally as genomic islands (GIs), and comprise a wide diversity of transmissible, transposable and replicative mobile genetic elements (MGEs) that differ even between closely related strains (Juhas et al., 2009). These elements contribute to genome diversification and evolution, with meaningful impacts on several diverse biological responses, including the acquisition and repurposing of catabolic pathways as well as the spread of antibiotic and virulence genes, among others (Frost et al., 2005).

One type of transmissible elements that integrate in the host genome, as a means of propagation and dispersal, are the Integrative and Conjugative Elements (ICEs). These elements share common characteristics with both plasmids and phages, with the distinctive feature between ICEs and other integrative mobile elements being their capability of self-transfer by means of conjugation (Burrus and Waldor, 2004; Wozniak and Waldor, 2010). Bacterial conjugation requires the biosynthesis and assembly of a specialized pilus by the donor cell [known as transferosome (de la Cruz et al., 2010)]. These conjugative pili pertain to the Type IV Secretion Systems (T4SS) and span the cellular envelope to contact the recipient cell and deliver the cargo DNA. T4SS are involved in several additional processes linked to conjugative transfer that, depending on the specific T4SS type (IV-A vs. IV-B) (Christie et al., 2005), include identification of a suitable recipient cell, signaling and processing of DNA for transfer initiation and/or dynamic assembly and retraction of the pilus (de la Cruz et al., 2010).

The archetypal T4SS includes the T-DNA transfer system of *Agrobacterium tumefaciens* (Christie, 2004) and the Tra-type systems of plasmids F, RP4, R388, and pKM101 (Ding et al., 2003; Christie et al., 2005; Juhas et al., 2008). In the case of the conjugative F-plasmid, a ∼30 kb-long transfer region encodes 33 *tra* genes (Frost et al., 1994). This system is able to produce a long and flexible pilus of 2–20 μm, with a diameter of 8 nm (Lawley et al., 2003). Best-characterized conjugative pili pertain to microorganisms thriving in less extreme environments, at neutral or physiologic pH and moderate temperatures. To date, little is known about the conjugation process and machinery in microorganisms from polyextreme environments, where high-oxidant and potentially hydrolytic conditions are the norm.

The acidithiobacilli are a diverse group of extreme acidophilic, Gram-negative, rod-shaped bacteria that obtain energy from inorganic electron donors, namely reduced and elemental forms of sulfur, ferrous iron and hydrogen. Recognized as an entirely new proteobacterial class, the *Acidithiobacillia* (Williams and Kelly, 2013), their taxonomy has been the focus of recent review and reclassification (Kelly and Wood, 2005; Hallberg et al., 2010; Hedrich and Johnson, 2013; Falagán and Johnson, 2016; Nuñez et al., 2017). Representatives of *Acidithiobacillus* occur in a broad range of natural (sulfur springs, acid rock drainage, etc.) and anthropogenic environments (ore piles, mineral concentrates, etc.), forming part of the microbial consortia used in the bioleaching of minerals for the recovery of metals of economic interest (Johnson, 2014). *Acidithiobacillus ferrooxidans* is by far the most widely studied member of this group (Quatrini and Johnson, 2018) and also the first of the species complex to be sequenced (Valdés et al., 2008). Since then, the genomes of several acidithiobacilli have been made publicly available, enabling comparative genomic studies of *Acidithiobacillus* species and strains (e.g., Zhang et al., 2016a,b). These studies have provided evidence of the occurrence of lateral gene transfer events in extreme niches (e.g., Acuña et al., 2013; Covarrubias et al., 2018). In particular, whole genome alignment of different strains of the iron oxidizing acidophilic model bacterium *A. ferrooxidans*, have revealed the presence of large mobile elements pertaining to each of the strains (Holmes et al., 2009; Levicán et al., 2009; Orellana and Jerez, 2011; Bustamante et al., 2014; Flores-Ríos et al., 2017). One of these elements, present in the type strain of the species (ATCC 23270^TY^) and named ICE*Afe*1, has all the diagnostic characteristics of an ICE (Bustamante et al., 2012). Recent studies have shown this element to be capable of excision out of the chromosome under normal and DNA-damaging growth conditions (Bustamante et al., 2012), in events directed by its own highly specific tyrosine recombinase (Castillo et al., 2017). To date, the ICE*Afe*1 element (or members of this MGE family) has only been identified in the *A. ferrooxidans*-type strain, even if conjugative genes used as markers in PCR-based screens of diverse strains have suggested a wider presence of the element in the taxon (Flores-Ríos et al., 2017). Altogether these facts suggest that this element is indeed active in conjugation and that the means by which it spreads is through the Tra-type T4SS encoded within the ICE.

A number of studies have been performed in recent years on the pili of *A. ferrooxidans*, due to the roles of these types of extracellular structures in different aspects of the biology of bacteria, particularly in mineral surface adhesion and colonization, twitching motility and electron transfer (Li et al., 2010, 2013; Li and Li, 2014). Yet, these functions are performed by a different kind of type IV pili known as Tfp-pili, and encoded by the *pil* gene cluster (Li et al., 2010, 2013; Li and Li, 2014), which do not have a role in conjugation. Type IV pili (Trb and Tra-type) involved in the conjugative transfer of DNA in these acidophiles, even if known to exist (Flores-Ríos et al., 2017) have not yet been proven functional.

To assess this, we have done a detailed bioinformatic analysis of the conjugative transfer region of ICE*Afe*1, evaluated its transcriptional expression and modeled the pilin protein and the multimeric structure derived from it. In addition, we have applied epifluorescence microscopy, laser-scanning confocal microscopy and immunomicroscopy (immunogold) to evaluate the protein level expression of the pilin and its extracellular localization in *A. ferrooxidans* cells. Our results prove that the ICE*Afe*1 encodes and expresses the minimal number of components to produce a functional T4SS, and that the pilin reaches the cell surface, and establishes a conjugative bridge between cells under growth conditions with both soluble and solid energy sources. These suggest the possibility of conjugative transfer between related microorganisms, in a bacterial group previously considered as recalcitrant to genetic modification, having impact in the genome plasticity of these acidophiles.

## Materials and Methods

### Bacterial Strains and Growth Conditions

The type strains *A. ferrooxidans* ATCC 23270^TY^ and *A. ferrooxidans* ATCC 53993 were grown at 30°C in modified 9K medium (pH 1.8) (Silverman and Lundgren, 1959) supplemented with 120 mM ferrous sulfate (FeSO_4_ × 7H_2_O), in modified 9K medium (pH 3.5) supplemented with 0.5% elemental sulfur (S^0^), or in MSM medium [3.0 g/l (NH_4_)_2_SO_4_, 3.2 g/l Na_2_SO_4_ × 10 H_2_O, 0.1 g/l KCl, 0.05 g/l K_2_HPO_4_, 0.5 g/l MgSO_4_ × 7 H_2_O, 0.01 g/l Ca(NO_3_)_2_], pH 2.5 supplemented with 5 mM tetrathionate (K_2_S_4_O_6_).

### Mitomycin C Treatment

Cells were grown in 9K medium supplemented with sulfur until late exponential-phase and collected by centrifugation. Then, the pellet was divided in two parts and each one was resuspended in 10 ml with 9K medium (pH 3.5) with sulfur. One culture was treated with 2 μg/ml mitomycin C (Sigma). Both cultures were incubated for 27 h at 30°C with agitation. Afterwards, RNA was extracted. All experiments were made in triplicate.

### General Nucleic Acids Techniques

DNA from *A. ferrooxidans* was isolated using a Wizard Genomic DNA Purification Kit (Promega) with the following modification for cell lysis: bacterial pellet was resuspended in Nucleic Lysis Solution, frozen at –80°C for 10 min and immediately heated at 80°C for 10 min. This procedure was repeated three times and then allowed to cool to room temperature (RT). The rest of the lysis protocol was performed as recommended by the manufacturer. RNA was isolated using TRIzol reagent (Invitrogen) from fresh bacterial pellet. The removal of genomic DNA from RNA preparations was carried out by digestion with DNase I (Fermentas). DNA and RNA quality were evaluated by 1.0% agarose gel electrophoresis and their concentrations were measured by absorbance at 260 nm in a NanoDrop 2000 Spectrophotometer (Thermo Fisher Scientific, Waltham, MA, United States).

### General PCR Techniques

PCR-based amplification of DNA sequences was performed using DreamTaq DNA Polymerase (Invitrogen) according to the protocol provided by the manufacturer. The cycling conditions were as follows: initial denaturation (95°C, 1 min), 30 cycles consisting of denaturation (95°C, 30 s), primer annealing [(at the estimated primer annealing temperature), 30 s], and extension (72°C, 1 min/kb); followed by a final extension step (72°C, 5 min). PCR products were visualized on 2% agarose gels stained with SYBR^®^ Safe DNA Gel Stain (Invitrogen). Real-time PCR reactions were performed in the Rotor-Gene Q PCR System (Qiagen) using the Kapa Sybr Fast^®^ (Sigma). The 20 μl PCR reactions contained 2 μl of a 1:100 diluted cDNA sample; 200 nM of each primer and 16 μl KAPA Master Mix. The cycling protocol was as follows: initial denaturation for 10 min at 95°C followed by 40 cycles of 3 s at 95°C, 20 s at 60°C; 1 s at 72°C. Fluorescence was measured after the extension phase at 72°C. The PCR products were subjected to a melting curve analysis that commenced at 52°C and increased at 0.5°C s^-1^ up to 95°C, with a continuous fluorescent measurement. Specific amplification was confirmed by a single peak in the melting curve. For each experimental condition, stationary-phase genomic DNA was extracted from two independent cultures. The reactions for each target gene were performed in triplicate and in the same PCR run. Amplification of DNA to generate the standard curve for qPCR was performed by the Kapa Sybr Fast^®^ (Sigma) according to the protocol provided by the manufacturer. Oligonucleotides used in this study for PCR and real-time PCR are listed in Supplementary Table 1.

### Production of Anti-pilin Serum

We designed several synthetic peptides for the TraA1 pilin, spanning most of the mature protein (86 aa) in order to produce anti-pilin serum. Five peptides were synthetized in GenScript^1^. A mix of the synthetic peptides (200 μg in total) was injected intraperitoneally (IP) to New Zealand rabbits to produce polyclonal antibodies against the pilin protein (anti-pilin serum). The rabbits were immunized four times (every 7 days) with the mix of synthetic peptides. Complete Freund’s Adjuvant (CFA) was used on the first injection and Incomplete Freund’s Adjuvant (IFA) in subsequent injections. The serum was used for pilin detection over *Acidithiobacillus*.

### Immunofluorescence Microscopy

Cells were grown until the exponential phase in MSM medium supplemented with tetrathionate. Cultures were collected by centrifugation at 1,000 × *g* for 20 min at RT. The cell pellets were first washed with MSM medium without tetrathionate for 5 m at RT and next with PBS pH 7.4. Then, cells were fixed by using 4% paraformaldehyde in PBS for 30 m at RT on obscurity. Fixed cells were rinsed and incubated with 3 μM DAPI for 20 m at obscurity (when using *Afe*Green1 the latter step was omitted). Subsequently, the cells were incubated with pre-immune serum or serum anti-pilin at a 1:1,000 dilution for 1.5 h in the absence of light. Finally, cells were washed with PBS and incubated with AlexaFluor 555 anti-rabbit at 1:2,000 dilution and immobilized on agarose 0.5% prepared on PBS for epifluorescence microscopy.

### Transmission Electron Microscopy

Cells were grown until the exponential phase in 9K medium supplemented with sulfur. Cultures were collected by centrifugation at 1,000 × *g* for 20 m at RT. Cells were washed first with 9K medium without sulfur at 1,000 × *g* for 5 m at RT and next with 100 mM sodium phosphate buffer (pH 7.2), before fixation using 3% glutaraldehyde and gentle mixing. Afterwards, cells were post-fixed in 1% (w/v) OsO4 for 1 h, dehydrated in ethanol series, and embedded in Epon 812 resin. Ultrathin sections were obtained using a Sorvall Porter Blum ultramicrotome, stained with uranyl acetate and lead citrate, and observed with a Phillips Tecnai 12 Biotwin TEM (Pontificia Universidad Católica de Chile) at an accelerating voltage of 80 kV.

### Immunogold Labeling

Cells were grown until the exponential phase in 9K medium supplemented with sulfur. Cultures were collected by centrifugation at 1,000 × *g* for 20 m at RT. Clean cell pellets (obtained as above) were fixed using a mix of 0.2% glutaraldehyde and 4% paraformaldehyde and gentle mixing. Afterwards, cells were washed with 0.2 M Tris–buffer (pH 7.2), dehydrated in ethanol series, and embedded in Poly/Bed 812 epoxy resin for ultra-thin sectioning. Ultrathin sections were obtained using a Sorvall Porter Blum ultramicrotome and placed on nickel grids. The grids were hydrated with 0.2 M Tris–buffer (pH 7.2) and blocked with immune buffer (1% BSA in 0.2 M Tris–buffer with 0.02% sodium azide). The primary antibody was diluted to 1:500 in immune buffer and incubated with the samples at 4°C for 16 h. The secondary antibody conjugated with 18 nm gold (Jackson Immunoresearch) was diluted at 1:20 in immune buffer and incubated with the samples for 2 h at RT. The grids were stained with 5% uranyl acetate for 5 min, rinsed with distillated H_2_O and observed with a FEI Inspect-F50 STEM (Universidad de Chile) at an accelerating voltage of 20 kV. Controls using pre-immune serum only and the secondary antibody only were applied to the cells to check for non-specific reactions.

### Gene Annotation, Curation and Gene Neighborhood Analysis

The ICE*Afe*1 sequence was obtained in FASTA format and entered to the RAST automatic annotation platform^2^. The results were compared with the available annotation of the *A. ferrooxidans* ATCC 23270^TY^ genome (NC_011761). Manual curation was performed when necessary. Linear DNA sequences in FASTA format were aligned using BlastN tools and visualized in Artemis ACT^3^. Promoters were predicted using the BPROM (Solovyev and Salamov, 2011) and NNPP (Reese, 2001) servers, considering intergenic regions between 50 and 500 pb. The Rho-independent terminators were predicted using ARNold (Gautheret and Lambert, 2001), FindTerm (Solovyev and Salamov, 2011), and RibEx (Abreu-Goodger and Merino, 2005) using default parameters.

### Heatmap Construction

Publicly available genomes of *Acidithiobacillus* (drafts and closed) were recovered form NCBI and re-annotated. ORFs were predicted using the GeneMarkS+ software with MetageneMark_v1mod model and used to construct a database with Makeblastdb. Candidate proteins were searched in this database using *A. ferrooxidans* ATCC 23270^TY^ Tra-system proteins as queries and BlastP as search algorithm with a cutoff *E*-value of 0.0001. To establish the amino acidic similarity (S) between query and candidate proteins, the SSEARCH software with the Smith-Waterman algorithm was used. Using the similarity matrix and the gplots v3.0.1 library from Rstudio a heatmap was derived. High amino acid similarity values were represented by warm colors (>60% S; >80% coverage) and low similarity values by cold colors. The dendrogram was constructed using Euclidian distance.

### Pilin Modeling

The secondary structure prediction of TraA1 and TraA2 were made using the JPRED (Drozdetskiy et al., 2015) and PSI-PRED (McGuffin et al., 2000) servers, with default parameters. The template identification was made with Psi-Blast and HHpred. For monomer and multimer modeling the template PDB^4^ 5LEG (at 3.5 Å resolution) was used. Multiple alignments between TraA1, TraA2 and template amino acid sequences were made by Clustal Omega (Chenna et al., 2003). Visualization and alignment analysis were made by Jalview software (Waterhouse et al., 2009). Comparative modeling of the TraA1/2 monomers and the respective multimers was done using Modeller v9.2 (Sali and Blundell, 1993) and STAMP software (Russell and Barton, 1992), and the 3D structure of PDB 5LEG as a template. All minimization and molecular dynamics simulations were performed using NAMD software (Phillips et al., 2005) and the CHARMM36 force field (Huang et al., 2017). Proteins were solvated with TIP3P water molecules and using counterions (Na^+^, Cl^-^) for system neutralization through VMD software (Humphrey et al., 1996). All simulations were performed at all-atom resolution for 5 ns at 310 K using an NPT ensemble. The evaluation of the molecular models was made analyzing the Ramachandran graphic (Ramachandran et al., 1963), using Rampage software. The Verify 3D method (Eisenberg et al., 1997) showed that all models are within the allowed limits of structured regions. The spatial analysis and protein visualization were made with VMD (Humphrey et al., 1996) and Pymol^5^ v2.0 (Schrödinger, NY, United States).

## Results and Discussion

### ICE*Afe*1 Encodes the Genes Required to Produce a Functional T4SS

**Figure 1:**
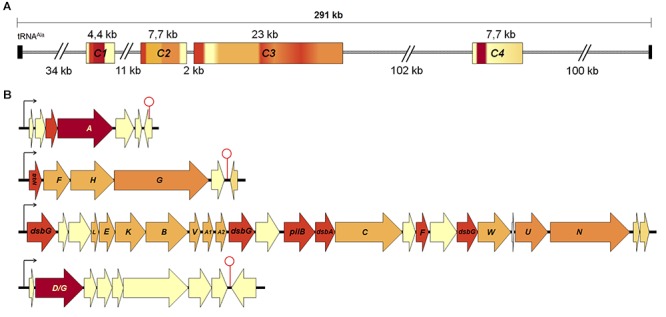
Organization of conjugative clusters present in the ICE*Afe*1. **(A)** Distribution of conjugative clusters encoded in ICE*Afe*1. The clusters were enumerated from 1 to 4 (C1 to C4) from the closest to tRNA^Ala^ onward. **(B)** Conjugative clusters organized in predicted operons and *tra* gene names are indicated in capital letters. Arrows and stem-loops represent predicted promoter and terminators, respectively. Proximal and distal black boxes in **(B)** indicate the tRNA repeats. The color gradient of each cluster in **(A)** corresponds to gene color coding in **(B)**. In red DNA processing functions, in orange mating pair stabilization functions, in pale orange pilus assembly functions, in dark orange accessory genes and in yellow hypothetical genes.

Twenty-one ORFs present in *A. ferrooxidans* ICE*Afe*1 were predicted to encode bacterial T4SS proteins potentially involved in the conjugative transfer of this genetic element. To assess this prediction, the ICE*Afe*1 was re-annotated and curated against updated databases (v 2018). Curation and updated functional assignment results for the T4SS are summarized in Table 1. Extensive annotation of the ICE*Afe*1 element can be found in Supplementary Table 2. Sequence similarity and motif/domain conservation analyses indicate that ICE*Afe*1 encodes eleven proteins required for pilus assembly, three proteins required for stabilization of mating pairs, two proteins required for conjugative DNA processing, and seven auxiliary proteins. All twenty-four structural and auxiliary T4SS genes are organized in four gene clusters–C1 to C4 (plus two conjugative genes outside these clusters)–spanning a total of 40 kb (Figure 1).

**Table 1:** Prediction of domains and subcellular location of T4SS_ICE_*_Afe_*_1_ components.

		Protein		Cellular	
ORF	Gene	size (aa)	Equivalence	localization	Motif / domain	Predicted function	*E*-value	CDD ID
AFE1047	*traA*	711	VirD2	C	Helicase/relaxase TraA	Relaxase	2.01E-27	TIGR02768
AFE1065	*trbN*	159	VirB1	C/P	Lytic transglycosylase	Pore generating at membrane	4.74E-13	pfam01464
AFE1066	*traF*	337	–	P	TraF	Pilus assembly	4.74E-72	pfam13728
AFE1067	*traH*	558	–	P/ME	TraH	Pilus assembly	2.48E-34	pfam06122
AFE1068	*traG*	1218	–	P/MI	TraG	Mating pair stabilization	8.98E-22	pfam07916
AFE1078	*traL*	92	VirB3	MI	TraL	Pilus assembly	1.69E-07	pfam07178
AFE1079	*traE*	204	VirB5	P/MI	TraE	Pilus assembly	1.49E-73	TIGR02761
AFE1080	*traK*	390	VirB9	P	TraK	Pilus assembly	2.19E-15	pfam06586
AFE1081	*traB*	543	VirB10	P	TraB	Pilus assembly	5.46E-12	PRK13729
AFE1082	*traV*	165	VirB7	P	TraV	Pilus assembly	6.74E-60	TIGR02747
AFE1084	*traA1*	135	VirB2	MI/EC	ND	Pilin	ND	ND
AFE1083	*traA2*	136	VirB2	MI/EC	ND	Pilin	ND	ND
AFE1087	*pilB*	396	VirB11	C	ATPase	Pilus assembly	1.00E-93	COG2804
AFE1089	*traC*	866	VirB4	MI	TraC / ATPase	Pilus assembly	5.37E-23	pfam11130
AFE1091	*traF*	184	–	MI/P	S26 signal peptidase	Signal peptidase	1.60E-06	TIGR02771
AFE1095	*traW*	422	–	MI/P	TraW	Pilus assembly	2.61E-11	TIGR02743
AFE1096	*traU*	416	–	P	TraU	Mating pair stabilization	1.56E-59	pfam06834
AFE1097	*traN*	1019	–	P/EC	TraN	Mating pair stabilization	9.46E-26	PRK12355
AFE1251	*traD*	619	VirD4	MI	TraD / ATPase	Coupling protein	2.19E-20	TIGR03743
AFE1235	*trbE*	222	VirB4	C	TrbE / ATPase	Pilus assembly	8.00E-07	COG3451
AFE1243	*traR*	104	–	C	C4-type zinc finger	Transcriptional regulator	4.00E-04	COG1734

Among the T4SS protein products identified in ICE*Afe*1, 68% are hallmarks of the Tra-type system found in F-like plasmids. The only exception is represented by the conjugative DNA relaxase, which is most similar to TraA of P-type plasmids (Lanka and Wilkins, 1995; Kopec et al., 2005; Yang et al., 2007). Typical domains distinctive of the P-type relaxases, including an N-terminal single strand exonuclease (COG0507) and a C-terminal helicase domain (pfam13538), were well conserved in the ICE*Afe*1-encoded relaxase, and were thus expected to fulfill the same role as TraI of F-type plasmids, nicking ICE*Afe*1 at its *oriT* (Datta et al., 2003) and unwinding the coiled circularized ICE prior to its conjugative transfer (Matson et al., 2001). Other chimeric arrangements have been reported previously in GIs (e.g., Ramsey et al., 2011). In addition to the T4SS gene orthologs, eighteen open reading frames encoding hypothetical and orphan genes interspersed between the *tra* genes in all four gene clusters were identified. Some of these hypothetical genes are well conserved in other acidithiobacilli and may represent novel ICE-T4SS-related functions requiring further experimental validation.

### The ICE*Afe*1 T4SS Components Occur in Other *Acidithiobacillus* Species Complex Members

**Figure 2:**
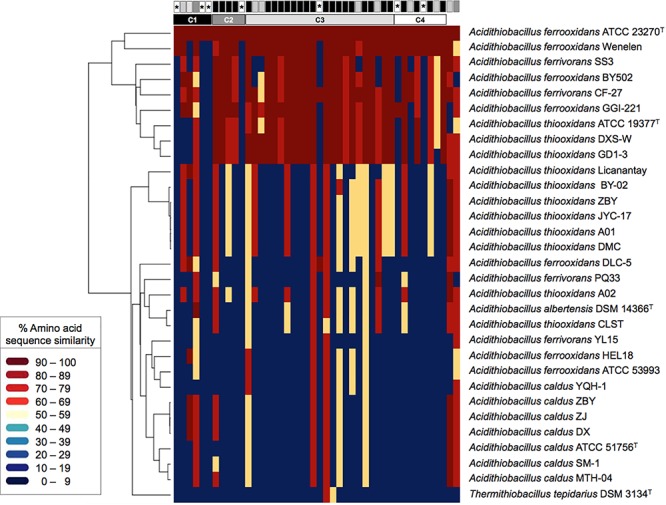
Heatmap of amino acid sequence similarity values for predicted proteins encoded within conjugative gene clusters in public *Acidithiobacilli* genomes. The genomes used are listed as follows (strain: accession) per species: *A. albertensis* (DSM 14663: MOAD01); *A. caldus* (ATCC 51756: NZ_CP005986; DX: LZYE01; MTH-04: LXQG01; SM-1: NC_015850; ZBY:LZYF01; ZJ: LZYG01); *A. ferrivorans* (CF27: CCCS02; PQ33: LVZL01; SS3: NC_015942; YL15: MASQ01); *A. ferrooxidans* (ATCC 23270: NC_011761; ATCC 53993: NC_011206; BY0502 : LVXZ01; DLC-5: JNNH01; GGI-221: AEFB01; Hel18: LQRJ01; YQH-1: LJBT01); *A. thiooxidans* (A01: AZMO01; A02: LWSA01; ATCC 19377: AFOH01; BY-02: LWRZ01; CLST: LGYM01; DXS-W: LWRY01; GD1-3: LWSC01; JYC-17:LWSD01; Licanantay: JMEB01; ZBY: LZYI01) and *Thermithiobacillus tepidarius* (DSM 3134: AUIS01). The genome of the *A. ferrooxidans* strain Wenelen was recovered from: http://biominingdb.cmm.uchile.cl/genomes/. Individual genes and gene clusters are represented by boxes above and their global conservation in gray-scale colors, from most conserved in black to least conserved in white. The asterisks indicate orphan genes. High amino acid similarity (S) values are represented by warm colors (>60% S) and low similarity values by cold colors (<30% S). Supporting alignments are available for download from Figshare at doi: 10.6084/m9.figshare.7538846.

To evaluate whether the ICE*Afe*1 T4SS is widespread in the taxon and the gene clusters and hypothetical genes linked to this element are conserved, publicly available *Acidithiobacillus* genomes sequences were searched using *A. ferrooxidans* ATCC 23270^TY^ *tra* gene products as queries. The amino acidic sequence similarity percentage values recovered for each genome (Supplementary Table 3) were plotted as a heatmap (Figure 2), using warm colors to display high amino acid sequence similarity levels (>60% S) and cold colors to denote low similarity or absence of potential orthologs (in dark blue). Supporting alignments in FASTA format for each *tra* gene product are available for download from Figshare at doi: 10.6084/m9.figshare.7538846.

Thirty-seven of the 44 *tra* genes profiled (84%) occurred in a set of nine *Acidithiobacillus* spp., representing 30% of the strains analyzed (Figure 2). These strains include *A. ferrooxidans* (3), *A. ferrivorans* (2) and *A. thiooxidans* (3) representatives. Average amino acidic similarity between the *tra*-gene products in this subset of strains was 79% S (Supplementary Table 3). Occurrence and conservation levels in three out of the seven recognized linages of the species complex suggests that this T4SS is functionally conserved and susceptible to conjugation between a fairly broad range of acidithiobacilli. Despite this fact, the Tra-type T4SS is much less widespread than the Trb-type T4SS [which has been reported to occur in 90% of sequenced strains (Flores-Ríos et al., 2017)], being completely absent in *A. caldus* and poorly represented in the *A. thiooxidans* genomes analyzed (30%).

Seven (out of 18) of the hypothetical proteins encoded by ORFs occurring in the *tra*-gene clusters of the ICE*Afe*1 (ORF1, ORF5, ORF6, ORF11, ORF23, ORF35, and ORF39) were not identified in the other acidithiobacilli genomes analyzed, even when using less stringent search criteria (blastp, tblastn, *E*-value: 0.01). Most of these proteins (encoded by AFE1045, AFE1048, AFE1049, AFE1069, AFE1086, AFE1250, and AFE1252) also lacked hits against the *nr* database, suggesting that these orphans do not correspond to true conjugative genes. A limited number of *tra* genes (*traA* relaxase, *trbN*, *traH*, *traB*, *traC*, *traF*, *traU*, *traN*, *traD*, *traR*, and *trbE*) and other accessory gene products (*pilB*, *dsbG*, *dsbA*) produced lower similarity hits in a larger set of strains (average 61.8% S; min. 48.9% S for ORF39 to max. 82.8% S for TraR), which actually correspond to *trb*-type orthologs present in these genomes. Altogether, the heatmap data revealed that the ICE*Afe*1 Tra-type conjugative system has spread among *Acidithiobacillus* species providing further support to its presumed functionality.

### The ICE*Afe*1 T4SS Gene Cluster Organization Resembles the Conjugative Cluster in Well-Known MGEs

**Figure 3:**
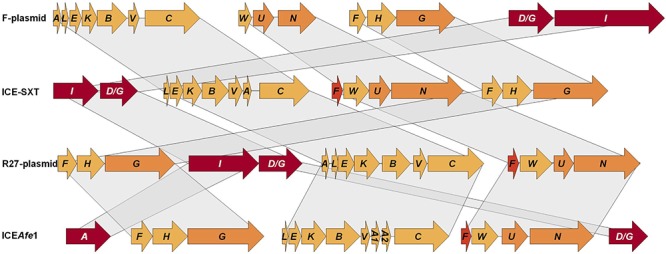
Relationship between conjugative clusters of ICE*Afe*1 and orthologs present in plasmids and other ICEs. Schemes for the conjugative gene clusters from non-acidithiobacilli microorganisms were modified from Frost et al. (1994) and Böltner et al. (2002).

The conjugative genes described above encode homologs of proteins previously shown to be involved in the transmission of ICEs as ICE^SXT^, ICE^R391^ (Böltner and Osborn, 2004) or plasmids as F and R27 (Frost et al., 1994), with similar genetic organization (Figure 3). These elements have four conjugative clusters with functions clearly identified: cluster I encodes for relaxase and coupling protein, involved in DNA-processing; cluster II encodes pilin and other proteins which are involved in pilus synthesis and assembly; cluster III and IV encode for proteins involved in pilus assembly and mating pair stabilization (Böltner et al., 2002). In this work, we identified four conjugative clusters in ICE*Afe*1, where clusters C1 and C4 encode the relaxase and the coupling protein, respectively, and clusters C2 and C3 encode proteins involved in pilin synthesis, and pilus assembly and stabilization (Figure 3). The cluster organization is similar between sequenced strains of the *Acidithiobacillus* species complex, supporting the idea that conjugative clusters have a common ancestor or that lateral gene transfer occurred between members of this bacterial group.

### ICE*Afe*1 T4SS Transcriptional Units Are Transcribed Under Basal and S.O.S. Conditions

The level of mRNA from predicted transcriptional units was evaluated experimentally using RT-PCR and RT-qPCR. RNAs were isolated from *A. ferrooxidans* ATCC 23270^TY^ mid-exponential aerobic 9K-Fe cultures (pH 1.8, 30°C) or 9K-S° cultures (pH 3.5, 30°C), and Mitomycin C (Mit C) treated cultures. This DNA alkylating agent (Mit C, 2 μg/ml) was used to induce DNA damage in 9K-S° grown cultures (mid log); conditions upon which other ICE elements excise out from their hosts’ chromosomes and activate the transcriptional expression of their genes (e.g., Beaber et al., 2004; Auchtung et al., 2005; Bellanger et al., 2007). Primers used are shown in Supplementary Table 1.

**Figure 4:**
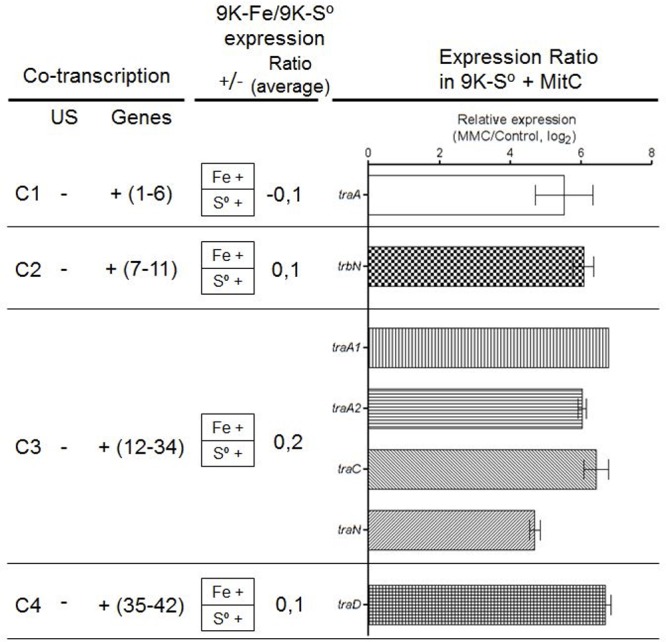
Expression levels of signature conjugative genes. **(Left)** Co-transcription assay per cluster (C1 to C4). **(Middle)** Expression ratio in Fe^2+^ or S^0^ media (data obtained from Quatrini et al., 2009). **(Right)** The log_2_ ratio of mRNA levels as determined by qRT-PCR from Mit C treated cell cultures versus control cultures (*traA* data obtained from Bustamante et al., 2012). Data were normalized to the expression level of *rpoC* gene. US, upstream gene. All experiments were performed triplicate.

RT-PCR evaluation of the *tra* genes encoded in the ICE*Afe*1 demonstrated transcriptional expression of all genes under the conditions tested and confirmed their organization as four independent transcriptional units of 4.7, 7.7, 23, and 7.7 kb, respectively (Figure 4 and Supplementary Figure 1). According to microarray data (Quatrini et al., 2009), these genes are not differentially expressed under optimal growth conditions, regardless of the energy source (Figure 4 and Supplementary Table 4). However, treatment of the 9K-S° grown cells with Mit C revealed a significant increase in the mRNA levels of selected marker genes present in each of the four operons with respect to the housekeeping *rpoC* gene, as assessed by quantitative RT-PCR analysis (Figure 4). Induction levels varied between 4-fold for the *traA/I* relaxase encoding gene (C1) to 7-fold in the case of the TraA pilin encoding gene paralog *traA1* (C3). Fold induction of these genes is consistent with the high protein expression levels expected for the pilin during mating pair pilus biosynthesis and assembly [e.g., ∼3,500 subunits for a 1 μm long pilus, as derived from Costa et al. (2016)]. The data reveal that all conjugative genes are transcriptionally active and that their levels are increased under SOS-response, and also that they are organized as independent operons.

### ICE*Afe*1 T4SS Encodes Two Structurally Conserved TraA Pilins

To assess the similarities and/or differences between the TraA1 and TraA2 pilin paralogs (in ATCC 23270^TY^/Wenelen strains) and the single TraA orthologs identified in other acidithiobacilli, predicted primary and secondary structures were compared in terms of their amino acidic properties (Supplementary Figure 2). The ICE*Afe*1 pilins TraA1 and TraA2 are 98.5% or 98.8% similar, depending on whether the full or the mature proteins (without the signal peptide) are cross-compared. Both proteins differ between each other in 12 residues (seven in the 49 aa signal peptide region; five in the mature pilin; Supplementary Figure 2B). All substitutions in the signal peptide are conservative (non-polar hydrophobic), while substitutions in the mature pilin include three conservative (non-polar hydrophobic V-I; polar uncharged hydrophilic T-N, S-T), two non-conservative changes (polar uncharged hydrophilic to non-polar hydrophobic, T-A; polar uncharged hydrophilic to polar positive hydrophilic T-K) and one extra C-terminal residue in TraA1 (non-polar hydrophobic A). When extending this comparison to other TraA orthologs found in the acidithiobacilli, additional substitutions were identified along the mature pilin. Most of these occur in the N-terminal alpha-helix and the C-terminal loop and are also conservative in nature (data not shown). However, additional charged residues (two to three positives; one negative) occurred in these orthologs (Supplementary Figure 2).

**Figure 5:**
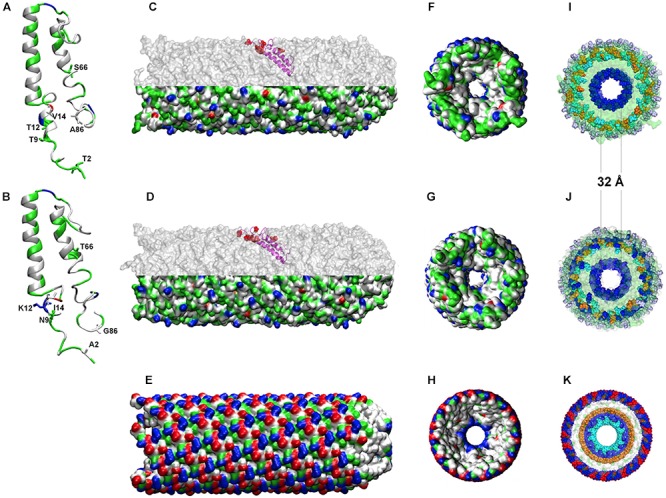
Predicted tertiary structures for TraA1 and TraA2 monomers and multimers. **(A,B)** Modeled TraA1 and TraA2 pilin monomers, respectively. **(C,D)** Modeled pilin multimers formed by TraA1 or TraA2, respectively. Upper half shows the position of one monomer in the channel. **(E)** 5LEG crystal-based model for *S. typhi* pilus. **(F–H)** Rear view of TraA1, TraA2, and 5LEG pilus, respectively, using van der Waals radio representation. **(I–K)** transversal view of the charged amino acid residues distribution of the three pili under comparison as above. The colors indicate the physicochemical properties as follows: blue, positive residues (Arg, cyan; Lys, blue; His, purple); red, negative residues (Asp, red; Glu, orange); green, polar residues and white, non-polar residues.

Given the availability of the recently crystalized F-type TraA pilin from *Salmonella typhi* plasmid pED208 (Costa et al., 2016), sharing ∼ 50% sequence similarity with both *A. ferrooxidans* TraA1 and TraA2, comparative modeling was pursued to reconstruct a likely 3D model for these acid-enduring pili. The mature TraA1 and TraA2 pilin monomers (i.e., without signal peptide) were modeled using the 5LEG PDB (50% similarity) as template (Figure 5A,B). With the exception of S66T, all substitutions observed in TraA2 locate in the proximal and distal ends of the monomer and are physically close in the 3D structure (Figure 5B). Structural analysis indicates that both alpha-helix-rich pilin monomers have similar physicochemical properties (pI TraA1 8.50; pI TraA2 9.53) and are predicted to adopt comparable 3D spatial conformations. The TraA1 and TraA2 monomers were thus used to model the multimer against the 5LEG PDB template for *S. typhi* TraA. The two 80-subunit multimers reconstructed for the ICE*Afe*1 pili are presented in Figure 5C,D. For comparison the pilus structure (5LEG) of *S. typhi* by Costa et al. (2016) is presented in Figure 5E.

The modeled pilins are predicted to establish the required inter-subunit interactions and to produce stable multimers. Orientation of the monomers within the 3D multimers shows that all conserved residues locate toward the channel lumen and the interfacial surfaces that conform the channel walls (Figure 5C,D and Supplementary Figure 2). The predicted internal lumen diameter for both TraA1- and TraA2-derived pili was 32 Å (Figure 5F,G), which is predicted to be sufficient for DNA to pass through the channel and comparable to the lumen diameter (28 Å) of *S. typhi F*-type pilus (Costa et al., 2016). Interestingly, in the context of the 3D multimer structure, most of the observed substitutions map to the exterior of the channel (Figure 5C,D, upper half, marked in red) and are therefore exposed to the extracellular medium. When compared against the *S. typhi* pilin crystal both TraA1 and TraA2 multimers showed a comparable distribution of charged amino acids toward the channel lumen (positive), conformed by a single layer of Lysine residues (K45) in *A. ferrooxidans* (Figure 5F,G,I,J) and a double layer of Arginine (R38) and Lysine residues (K41) in the case of *S. typhi* (Figure 5H,K). However, toward the exterior of the pili a dramatic change in the number and distribution of charged residues is apparent between the neutrophile and acidophile models (Figure 5C,D lower half and Figure 5E). Exposed alternating positive (Lysine) and negative (Aspartic acid) charges of *S. typhi* pilus (5LEG) are replaced by only partially exposed alternating positive (Arginine and/or Lysine) and negative (Glutamic acid) charges in the *A. ferrooxidans* models (surrounded by 10% more polar and non-polar residues). Remarkably, both TraA1 and TraA2 multimers have an additional outer layer of basic Histidine residues, which might act as a protective barrier against the high proton concentrations in the acidic milieu (Figure 5I,J). This observation is coherent with the net surface charge prediction (of +6) for both for TraA1 and TraA2 pili, and which contrasts with *S. typhi* pilus’ net charge of zero. These results are in line with previous reports, which indicate that extracellular or external membrane proteins of extreme acidophiles tend to be more basic and/or more hydrophobic than their neutrophilic counterparts (e.g., Ramírez et al., 2002; Quatrini et al., 2005; Manchur et al., 2011), probably reflecting an adaptive variation related to protein stabilization at low pH (Duarte, 2013).

### The ICE*Afe*1 T4SS Pili Is Produced in *A. ferrooxidans*^TY^ Cells

**Figure 6:**
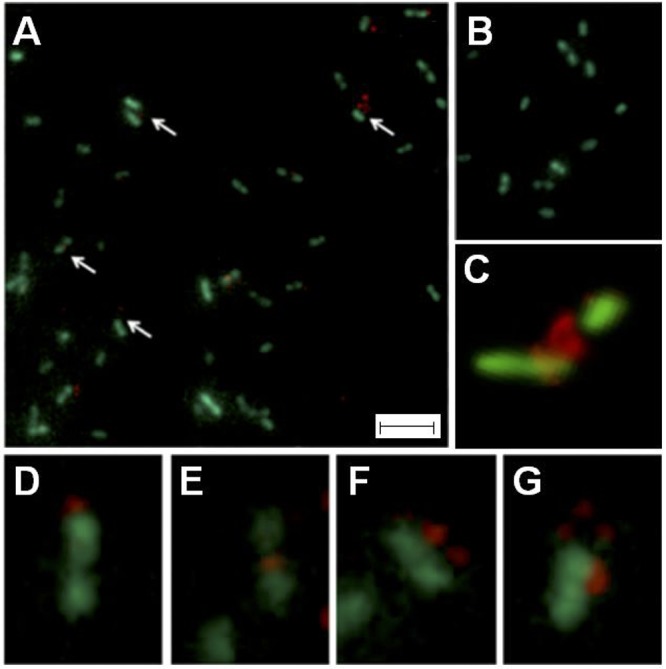
Visualization of pilus in *A. ferrooxidans^Afe^*^Green1^ cells by epifluorescence microscopy. **(A)** Cells (green) incubated with anti-pilin serum. **(B)** Cells (green) incubated with pre-immune serum. **(C)** Confocal microscopy image showing pilin-specific mark (red) in the interface between two cells. Pilin-specific marks are indicated with white arrows. **(C–G)** Magnifications of field sections of **(B)**. For **(A,B)** bar: 8 μm; for **(C–G)** bar: 2 μm.

To demonstrate whether the ICE*Afe*1 predicted conjugative bridge is expressed and exported to the bacterial surface, antibodies were raised against five synthetic peptides spanning the entire length of the mature TraA1 pilin protein, including conserved and variable regions between TraA1 and TraA2. This ICE*Afe*1 T4SS pilin-specific serum was used in combination with a red fluorescent secondary antibody (AlexaFluor 555) to detect the presence of the protein extracellularly in cells from mid-exponential 9K-S° liquid cultures of *A. ferrooxidans*. The recombinant strain *Afe*Green1 derived from *A. ferrooxidans*, ATCC 23270^TY^ expressing the cytoplasmic green fluorescent protein GFP, was used to visualize bacterial morphology. Samples were visualized by epifluorescence microscopy and confocal laser scanning fluorescent microscopy (Figure 6).

In all samples incubated with the anti-pilin serum analyzed (∼1,000 cells, experimental triplicate, technical triplicate), distinctive red spots were observed in one third of the cells (Figure 6A), while the control experiment lacked these signals (Figure 6B). The image analysis revealed that the number (one to three) and relative location of the red mark varied (with respect to the cells), as exemplified by the magnifications presented in Figure 6D–G. Of the cells showing immunoreaction with the pilin-specific antiserum, 60% had only one red spot located either in the mid-region (64.9 ± 5.0), in the apical region (26.1 ± 7.6) or between cells (15.8 ± 5.7). These results are in agreement with reports on other conjugative systems (Yan and Taylor, 1989; Lai et al., 2000; Carter et al., 2010; Saisongkorh et al., 2010). Location of the pilin-specific mark between cells was confirmed using confocal laser scanning fluorescent microscopy (Figure 6C).

**Figure 7:**
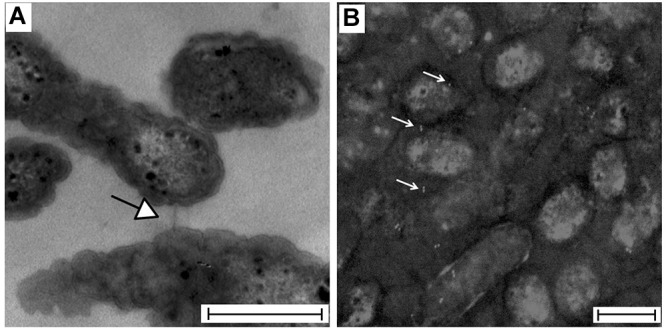
Visualization of *A. ferrooxidans*^TY^ cells by transmission electron microscopy. **(A)** TEM preparation showing an intercellular channel connecting two bacteria. **(B)** Immunogold showing positive signal for pilin (white) intercellularly or on the bacterial surface of Mitomycin C-treated cells (gray scale). Bar: 500 nm. The image was black-white inverted to improve the contrast.

Polymerization of the TraA1/TraA2 protein into a pili-like structure on the cellular surface of *A. ferrooxidans* was assessed through transmission electron microscopy (Figure 7A). While pili-like structures were readily observed in 9K-S°-grown *A. ferrooxidans* cells, these were seldom detected in cells grown with soluble tetrathionate as the energy source (data no shown). To further support the nature of the observed pili-like structures, 9K-S°-grown *A. ferrooxidans* cell cultures were processed for immunoelectron microscopy. The grids incubated with the ICE*Afe*1 T4SS-specific anti-pilin primary antibodies showed positive immunogold labeling of intercellular structures (Figure 7B). Sample preparations that had not been in contact with the anti-pilin primary antibodies showed no immunogold reaction (Supplementary Figure 3).

Both epifluorescence microscopy and immunogold assays performed herein confirmed that an extracellular structure composed of TraA1-like pilin is produced by *A. ferrooxidans* ATCC 23270^TY^ cells.

## Conclusion

In this work, the Tra-type T4SS encoded in *A. ferrooxidans* ATCC 23270^TY^ ICE*Afe*1 was characterized in terms of its organization, conservation, expression and mating bridge formation capacity. Thirty-seven predicted ORFs organized in four gene clusters, spanning ∼40 kb of the ICE, encode the required components to synthesize and stabilize the conjugative pilus, together with the genes required for DNA processing and relaxosome-mediated DNA transfer, all of which are transcriptionally active. A number of hypotheticals encoding potentially novel conjugation-related functions were also pinpointed. Most of the *tra* gene products encoded in the ICE*Afe*1 are conserved in terms of amino acid sequence similarity (>74% S on average) in 30% of the sequenced acidithiobacilli analyzed. Yet, these genes retain the characteristic variability signature of horizontally transferred genes. Occurrence of these *tra* genes in strains from distant geographical origins pertaining to three out of the seven recognized lineages of the species complex provides proof of the active spread of the Tra-type T4SS systems (and likely also their carrier MGEs).

Presence of two Tra-type pilin encoding genes was infrequent, both in sequenced acidithiobacilli and other neutrophilic bacteria, with only one additional example (*A. ferrooxidans* strain Wenelen) apart from that of strain ATCC 23270^TY^. The TraA1 and TraA2 pilins encoded in ICE*Afe*1 are conserved in primary and secondary structures and are predicted to adopt similar 3D spatial conformations to their closest crystalized ortholog (5LEG), despite a number of substitutions toward the proximal and distal ends of each protein. According to modeling-based analyses performed herein, both pilins can potentially produce stable multimers, with a channel lumen diameter and charge distribution that is compatible with conjugative DNA transfer and an exposed surface that shows signatures of adaptation to the acidic milieu. All conjugative genes proved to be transcriptionally active under optimal growth conditions for the species, and their levels increased dramatically in response to Mitomycin C treatment, a DNA damage elicitor that has acknowledged stimulatory effects on excision rates and gene expression of other ICEs. In addition, using a tailor-made pilin-antiserum against ICE*Afe*1 T4SS TraA pilin, and both epifluorescence and immunogold electron microscopy, the presence of the conjugative pili on this microorganism’s cell surface was demonstrated.

Together, these results demonstrate that the ICE*Afe*1 T4SS is phylogenetically conserved within the taxon, is expressed at mRNA and protein levels *in vivo* in the *A. ferrooxidans* type strain, and produces a pili-like structure of extracellular and intercellular localization in this model acidophile. The data constitute the first demonstration of the production of conjugative pilus in *Acidithiobacillus* representatives and provides support for the occurrence of DNA transfer in acid milieus.

## Author Contributions

RQ and OO conceived and designed the study. RF-R and SV performed the experiments. RF-R, AM-B, CP-B, and MA-S performed the bioinformatics analyses. RF-R, OO, and RQ analyzed and discussed the data. RQ and RF-R wrote the paper. All authors have read and approved the final manuscript.

## Conflict of Interest Statement

The authors declare that the research was conducted in the absence of any commercial or financial relationships that could be construed as a potential conflict of interest.

## Footnotes

1 www.genscript.com

2 http://rast.nmpdr.org/

3 http://www.sanger.ac.uk/resources/software/act

4 https://www.rcsb.org

5 https://pymol.org/2/
