# Supplementary material for: The Type IV Secretion System of ICEAfe1: Formation of a Conjugative Pilus in Acidithiobacillus ferrooxidans
Source: Front Microbiol. 2019 Feb 5;10:30. doi: 10.3389/fmicb.2019.00030 (PMC6370655; doi:10.3389/fmicb.2019.00030)
Supplement: Supplementary file 5 [file Table_5.docx]

Supplementary Material

**The Type IV Secretion System of ICE*Afe*1: formation**

**of a conjugative pilus in *Acidithiobacillus ferrooxidans***

**Flores-Ríos R^a,b^, Moya-Beltrán, A^a.c^, Pareja-Barrueto C^c^, Arenas M^d^, Valenzuela S^a^,**

**Orellana O^b^, Quatrini R^a^.**

* Correspondence

Dr. Raquel Quatrini

[rquatrini@cienciavida.org](mailto:alejandra.giaveno@probien.gob.ar%3e)

Dr. Omar Orellana

[oorellan@med.uchile.cl](mailto:oorellan@med.uchile.cl)

# Supplementary Figures and Tables

## Supplementary Tables

**Supplementary Table 1.** Primers used in this paper.

XLS file

**Supplementary Table 2**. Annotation of ICE*Afe*1 genes.

XLS file

**Supplementary Table 3**. Amino acidic sequence similarity values for proteins encoded in conjugative gene clusters in public *Acidithiobacilli* genomes.

XLS file

**Supplementary Table 4**. Log_2_ relative level of expression in *A. ferrooxidans*^TY^ cells grown on different energy substrates.

XLS file

## Supplementary Figures

**Supplementary Figure 1**. Operon analysis of conjugative gene clusters by RT-PCR.

PDF file

**Supplementary Figure 2**. Comparison of TraA1 and TraA2 pilin primary and secondary structures. (A) Protein identity between pilin TraA1 and TraA2 of the *A. ferrooxidans* ATCC 23270 ICE*Afe*1 versus *A. ferrooxidans* Wenelen and other acidithiobacilli. Identity values for complete and mature pilin (in parentesis) are shown. (B) MAFFT-based amino acidic sequence alignment of mature TraA-like pilins from *A. ferrooxidans* ATCC 23270 ICE*Afe*1, other acidithiobacilli and *S. typhi* (5LEG). The secondary structures were predicted using Jpred. Hydrophobicity index and polarity were calculated using Geneious v11.0.2. Amino acids in the alignment are colored according to their polarity as follows: yellow, non-polar; green: polar, uncharged; red: polar, acidic; blue: polar, basic. Amino acids in the logo above are colored according to their hydrophobicity index as follows: red, hydrophobic; blue, hydrophilic.

PDF file

**Supplementary Figure 3**. Original images for immunogold to *A. ferrooxidans*^TY^ treated with Mit C. (A) Cells incubated with pre-immune serum. (B) Cells incubated without anti-pilin serum.

PDF file
