# Supplementary figures and images for: The Type IV Secretion System of ICEAfe1: Formation of a Conjugative Pilus in Acidithiobacillus ferrooxidans
Source: Front Microbiol. 2019 Feb 5;10:30. doi: 10.3389/fmicb.2019.00030 (PMC6370655; doi:10.3389/fmicb.2019.00030)

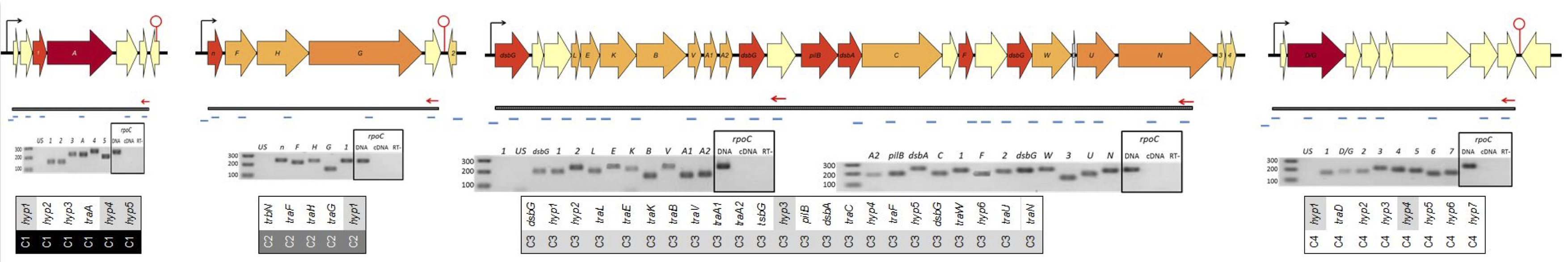

Supplement: Supplementary file 6 [file Image_1.TIFF]

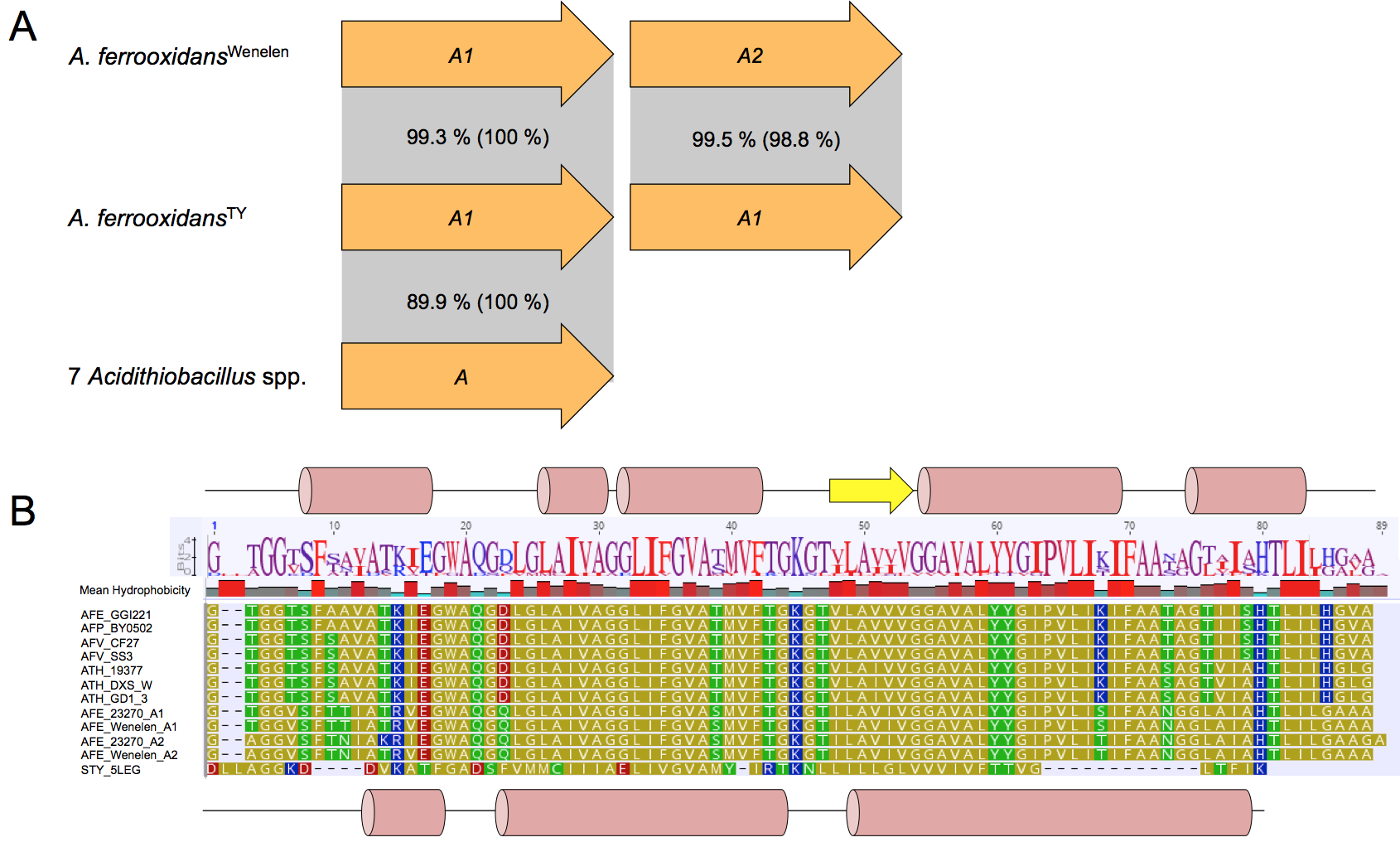

Supplement: Supplementary file 7 [file Image_2.TIFF]

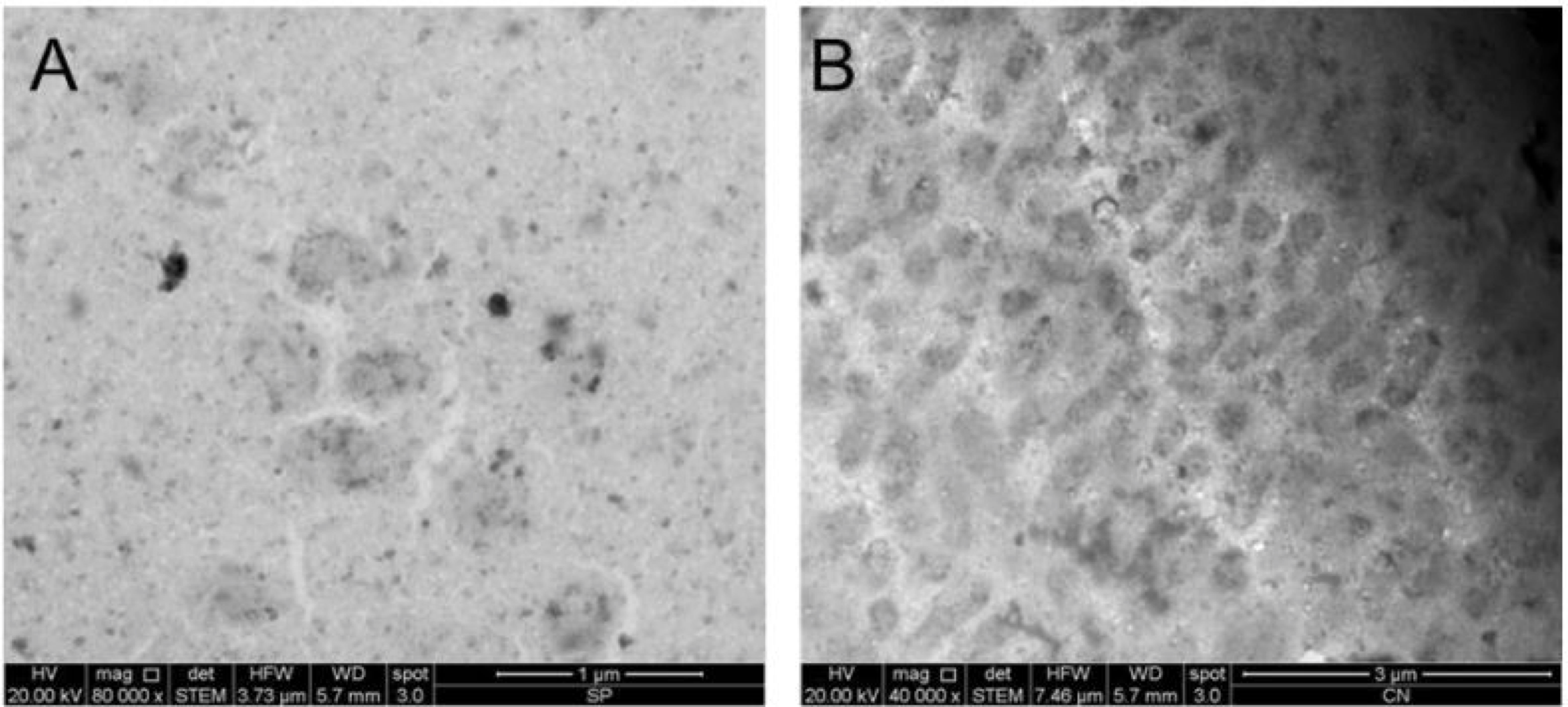

Supplement: Supplementary file 8 [file Image_3.TIFF]
